# Which Clinical Reasoning Models Are Used by Physiotherapists, Occupational Therapists and Selected Allied Health Professionals? A Scoping Review

**DOI:** 10.1002/hsr2.73227

**Published:** 2026-10-08

**Authors:** Henry Schultz, Rosa Virgara, Ellen O'Callaghan, Ali Aguilar, Kristin Graham, Saravana Kumar

**Affiliations:** ^1^ School of Allied Health and Human Performance Adelaide University Adelaide Australia; ^2^ Alliance for Research in Exercise, Nutrition and Activity Adelaide University Adelaide Australia; ^3^ IIMPACT in Health, School of Allied Health and Human Performance Adelaide University Adelaide Australia

**Keywords:** allied health, allied health personnel, clinical decision‐making, clinical reasoning, healthcare

## Abstract

**Background and Aims:**

Clinical reasoning is a critical skill for Allied Health professionals, enabling informed decision‐making in patient care. While a wide variety of clinical reasoning models are taught across disciplines, it remains unclear which models are frequently applied in practice. The scoping review aimed to map the literature on clinical reasoning models used by allied health professionals.

**Methods:**

This review was informed by Joanna Briggs Institute's framework for scoping review. Searches were conducted across Medline, Embase, Emcare, SCOPUS, CINAHL, the Cochrane Library and gray literature sources in January 2024. Results were screened in duplicate and data were descriptive synthesized and summarized the clinical reasoning approaches reported in the included studies.

**Results:**

Forty studies were included, spanning clinical reasoning literature from physiotherapy, occupational therapy, psychology, podiatry, speech pathology, and dietetics; no studies were identified for social work or exercise physiology. Hypothetico‐deductive reasoning and pattern recognition were the most commonly reported models of clinical reasoning across the included studies, while three track reasoning and dual‐process theory also featured across some professions. Seven additional models were also reported, though these were documented as isolated instances.

**Conclusion:**

While several clinical reasoning models are used by Allied Health professionals in practice, only a handful are commonly reported in the literature, particularly within physiotherapy and occupational therapy. This is despite Allied Health curricula and training programs drawing on a much broader range of models. Evidence for other Allied Health professions remain limited, highlighting a need for further research to determine whether these patterns hold more broadly. These findings suggest a need to reassess the teaching and application of clinical reasoning models within the professions studied. Further research, particularly with underrepresented Allied Health professions, can help identify preferences and drivers influencing model use in practice.

## 1 Background

Clinical reasoning describes the reflective process of gathering, analysing, and evaluating patient information to understand the person, environment, and/or presenting problem [1]. Although literature exploring the concept is primarily focused on the diagnostic process [2], clinical reasoning is continually undertaken by all health professionals to guide clinical practice and inform clinical decisions [3]. All health professionals use clinical reasoning when treating patients, albeit using varying models and styles [4]. It is context dependent, and most effective when considering patient factors, clinicians’ own abilities (i.e. manual skills, knowledge) and situational factors such as the practice setting [5]. While clinical reasoning is routine in practice, there are ongoing knowledge gaps on this topic. These include a limited understanding of which clinical reasoning models and frameworks are used by which disciplines [6] and whether one model is superior to another [7].

Much of clinical reasoning literature is based on medical, nursing, or health educational contexts [8]. Consequently, clinical reasoning has traditionally been conceptualized within a biomedical model of care [9]. The biomedical model is symptom‐focused and paternalistic, typically positioning the patient as passive recipients of care and interventions [9]. It conceptualizes health primarily as absence of disease and places limited emphasis on client‐centered care [9]. Within this model, clinical reasoning is primarily used for diagnostic purposes [10], whereas contemporary clinical reasoning models consider a broader range of factors such as patient contexts and experiences [11]. The biopsychosocial model [12], which is increasingly preferred [13], provides a more holistic approach by recognizing how a patient's health condition interacts with their cultural, physical and social environments [14].

Categorizing different styles of clinical reasoning has sought to enhance its transparency and explicitness of clinical reasoning among Allied Health professionals. Edwards et al. [2] identified several clinical reasoning styles that provide frameworks through which clinicians seek, interpret, act on, and disseminate clinical information to inform clinical decisions. Two main styles for clinical reasoning are narrative and diagnostic reasoning [15]. While narrative reasoning involves framing clinical problems in the unique contexts of the patient [15], diagnostic reasoning considers functional limitations more generally. Diagnostic reasoning is grounded in assumptions of a biomedical model, in which clinical problems are viewed as well‐defined, predictable, and objective [16]. In contrast, narrative reasoning aligns more closely with the biopsychosocial model, recognizing that clinical factors are context‐dependent and unique to each individual. Other reasoning styles include collaborative, procedural, teaching, interactive, predictive, and ethical reasoning. Edwards et al. [2] proposes that these styles lie along a continuum between narrative and diagnostic styles of clinical reasoning, and may be used by Allied Health professionals, as appropriate, to address different types of clinical problems. These reasoning styles underpin the use of clinical reasoning models, which provide structured and systematic approaches to clinical reasoning by describing the internal and external factors that Allied Health professionals consider when solving clinical problems.

Allied Health professionals constitute the second largest health workforce in Australia [17] and work across acute, sub‐acute, post‐acute, community based and primary health settings. Historically, Allied Health has been defined by the exclusion of medical and nursing professions, although there have been ongoing attempts to categorize and group different Allied Health disciplines [18]. Turnbull et al. [18] categorized Allied Health into therapeutic, diagnostic, and technical, scientific, and complementary services. However, to date, there is no agreed definition of Allied Health [19]. An environmental scan undertaken by Nancarrow et al. [20] identified 27 different Allied Health disciplines, while other systematic reviews identify smaller groupings (5 to 10) [19, 21, 22]. Despite this diversity, Allied Health disciplines share a focus on preventing, identifying, and treating a wide range of conditions and illnesses based on their specialist, non‐medical training area. For example, occupational therapists are specifically trained and specialized in managing activities of daily living [23]. Allied Health professionals also commonly work collaboratively with multidisciplinary teams, which may include physiotherapists, occupational therapists, speech therapists, podiatrists, to address patients’ needs holistically [24]. For the purposes of this review, allied health was defined as physiotherapy, occupational therapy, social work, nutrition and dietetics, speech therapy, exercise physiology, psychology, and podiatry. These disciplines were selected as those Allied Health professions with an established literature base examining broader clinical reasoning processes within patient assessment, diagnosis, and management and central to the research question. Studies involving other professions also classified as Allied Health (e.g., radiography, optometry) were therefore excluded, and this scope is reflected in the eligibility criteria described below.

While clinical reasoning is ubiquitous across health professions, individual disciplines have developed clinical reasoning models to suit their specific requirements [25]. In occupational therapy, Fleming [26] developed the Three Track Reasoning Model, which describes the rapid and dynamic drawing from procedural, interactive, and conditional reasoning in resolving clinical problems. In contrast, Jones et al. [27] identified clinical reasoning in physiotherapy literature as being hypothesis‐oriented, particularly utilizing hypothetico‐deductive reasoning. However, it remains unclear how these models are applied in practice. Much of the clinical reasoning literature has focused on teaching and learning of clinical reasoning, rather than how clinical reasoning is enacted by Allied Health professionals [6, 28, 29]. Furthermore, the existing Allied Health clinical reasoning literature has focussed on a limited number of disciplines and practice settings [30]. In a systematic review of clinical reasoning among student health professionals in placement and simulation settings, Brentnall et al. [31] found limited evidence of multidisciplinary clinical reasoning within Allied Health research, and highlighted inconsistencies in the terminology used to describe clinical reasoning.

While efforts have been made to investigate how clinical reasoning is taught to student Allied Health professionals [32], no research to date has synthesized the literature on clinical reasoning models used by Allied Health professionals in practice settings. Therefore, this scoping review aimed to map the literature on clinical reasoning models used by Allied Health professionals in practice.

## 2 Methods

### 2.1 Review Question

What clinical reasoning models are used by Allied Health professionals across physiotherapy, occupational therapy, social work, nutrition and dietetics, speech therapy, exercise physiology, psychology, and podiatry in acute, sub‐acute, post‐acute, community, and primary healthcare settings?

### 2.2 Study Design

A scoping review was selected because it offers a comprehensive approach to exploring nuanced and complex concepts that have not yet been extensively examined. This methodology enables the systematic collection, mapping, and synthesis of a wide range of existing literature, providing a broad understanding of the topic while identifying key themes, gaps, and potential directions for future research. It is particularly valuable for emerging areas where existing evidence is scattered or incomplete, making it ideal for capturing the full scope of relevant studies and insights. The review was undertaken according to the JBI approach for evidence synthesis [33] to ensure rigor and a structured framework and reported using the guidelines of the Preferred Reporting Items for Systematic Reviews and Meta‐Analyses Scoping Review (PRISMA‐ScR) [34]. The protocol was previously registered with the Open Science Framework (OSF) https://doi.org/10.17605/OSF.IO/ZJK6M.

### 2.3 Eligibility Criteria

A Population, Concept, Context (PCC) framework was used to inform the research question and the search strategy by establishing eligibility criteria (Table 1). Given the inconsistent terminology used across the clinical reasoning literature, the term clinical reasoning model was used in this review as an umbrella term encompassing conceptual approaches that explicitly describe, explain, structure, or guide the process of clinical reasoning. These included constructs referred to by original authors as models, theories, frameworks, reasoning approaches, or reasoning strategies, provided they described an identifiable approach to clinical reasoning rather than an isolated cognitive skill, educational technique, assessment method, or clinical competency. Studies were included if they involved one or more of the following allied health disciplines: physiotherapy, occupational therapy, social work, nutrition and dietetics, speech therapy, exercise physiology, psychology, or podiatry. Studies were excluded if they exclusively involved allied health professions outside this scope (e.g., medical radiation, optometry). In line with requirements for a scoping review, *all* relevant existing literature sources were included, including journal articles, theses, and gray literature. No year or language limits were applied, consistent with best practice in review methodology.

**Table 1:** Summary of eligibility criteria for considered studies.

	Inclusion criteria	Exclusion criteria
Population of interest	Allied health disciplines including: ◦ Physiotherapy, Occupational therapy, Social Work, Nutrition and Dietetics, Speech therapy, Exercise Physiology, Psychology, and Podiatry. Equivalent disciplines in foreign countries	Disciplines which undertake primarily diagnosis (e.g., medical radiation/radiography) or prescription (e.g., optometry) ◦ Professions such as radiography and optometry were excluded due to the predefined scope of this review, which focused on Allied Health professions with an established literature base examining broader clinical reasoning processes within patient assessment, diagnosis, and management. Non‐Allied Health professionals Students and educators in Allied Health professions
Concept of interest	Models of clinical reasoning applied by Allied Health professionals. ◦ Defined as: structured, systematic approaches to understanding and addressing clinical problems. ◦ Can draw from one or several clinical reasoning styles e.g., diagnostic reasoning to provide Allied Health professionals with a structured, applicable framework through which they conduct clinical reasoning.	Studies that focused on clinical reasoning as an unstructured process, clinical decision‐making analysis, or clinical reasoning as a neurobiological process. Clinical reasoning models applied by non‐Allied Health professionals. Clinical reasoning approaches which are not defined as models Clinical reasoning models which are purely theoretical and without practical application in practice.
Context of interest	Healthcare practice settings where Allied Health professionals practice ◦ Encompasses acute, sub‐acute, post‐acute, community based and primary health settings where Allied Health professionals deliver services to patients.	Academic institutions focused on clinical reasoning research instead of clinical reasoning practice. Studies that focused on algorithms or tools to assist with or evaluate clinical reasoning.
Language of publication	Publications in English, or non‐English publications with an available English translation	Sources published in languages other than English with no English translation available.
Source type	Published literature, including peer‐reviewed articles and books. Gray literature, including organizational reports and unpublished theses.	Opinion pieces, abstract, letters, editorials, reviews and commentaries

### 2.4 Information Sources

A comprehensive search of OVID Medline, OVID Embase, OVID Emcare, EBSCOHost CINAHL, SCOPUS and the Cochrane Library was undertaken on 25/1/2024. MEDLINE was searched via Ovid rather than through the PubMed interface, as the former provides equivalent core content with more advanced search functionality (e.g., subject heading mapping, proximity operators). Search strategies were customized to suit each database for relevant headings and wildcards. The search syntax of various databases are presented in Appendix A. For gray literature, Google Scholar and Trove were searched (Appendix B). Pearling by examining reference lists of included sources were screened for further potentially eligible studies. The search strategy was created in collaboration with an academic librarian.

### 2.5 Literature Selection

The primary author (H.S.) conducted the searches and imported the references into EndNote 20. Citations were then exported into Covidence review management software for screening and de‐duplication. Each article's title and abstract were independently screened in duplicate by the primary author (H.S.) and a secondary author (R.V., E.O., A.A., K.G., or S.K.). Disagreements were resolved through discussion between the primary author (H.S.) and a secondary author (R.V.) and did not require a third author. Each full‐text article was subsequently screened for inclusion by the primary author (H.S.) and a secondary author (S.K.). Reasons for exclusion were recorded at full text screening stage. A PRISMA flow chart was created detailing number of studies and reasons for exclusion.

### 2.6 Data Extraction

As recommended by JBI [33], a customized Excel spreadsheet was developed to table the data extraction (Appendix C). The data extraction form was piloted with six articles (approximately 10%), to ensure comprehensiveness and consistency between authors [35]. The remaining articles were extracted by the primary author (H.S.). Study characteristics including author(s), publication date, country, study design, information gathering methods, Allied Health professionals, and clinical reasoning model and its development and features were extracted. Extracted data was analysed by the primary author (H.S.) and refined through consultation with other authors (S.K., A.A., E.O., K.G., R.V.). Consistent with the objectives of this scoping review, critical appraisal of included sources was not undertaken. The aim of this review was to map and describe the clinical reasoning approaches reported in the Allied Health literature rather than assess methodological quality, effectiveness, or comparative outcomes of individual approaches.

### 2.7 Synthesis of Results

Owing to the nature of the data, a descriptive synthesis of the literature [36] was undertaken, The descriptive synthesis summarized the clinical reasoning approaches reported in the included studies. Data extraction focused on the identification and characteristics of clinical reasoning approaches, including the terminology used by study authors, conceptual description, allied health profession, and frequency of reporting. Where different terms described the same conceptual approach, terminology was standardized to the most commonly used descriptor while retaining the terminology reported in the original publication. The unit of analysis was the included publication. Where a single publication reported multiple clinical reasoning approaches, each approach was extracted and counted separately. Initial data extraction and classification were undertaken by the primary author (HS), with classifications reviewed and discussed with the remaining authors (SK, AA, EO, KG, RV). Any disagreements regarding classification were resolved through consensus.

## 3 Results

### 3.1 Search Results

The database search yielded 13,991 citations, after removal of duplicates 7802 remained. The titles and abstracts of these citations were screened, of which 7490 did not meet the inclusion criteria. Five of the remaining 312 citations were unable to be located. 307 citations were screened in full text, 267 were excluded for various reasons: focused on clinical decision‐making (*n* = 101), wrong study design (*n* = 66), identified no clinical reasoning models (*n* = 52), conference proceedings (*n* = 16), tools/algorithms to assist with clinical reasoning (*n* = 12), focused on the wrong population (*n* = 8), practice guidelines (*n* = 4), set in an education setting (*n* = 4), or theoretical clinical reasoning models with no evidence for clinical use (*n* = 4). The final number of studies included in the review was 40. Figure 1 shows the process in a PRISMA diagram.

**Figure 1:**
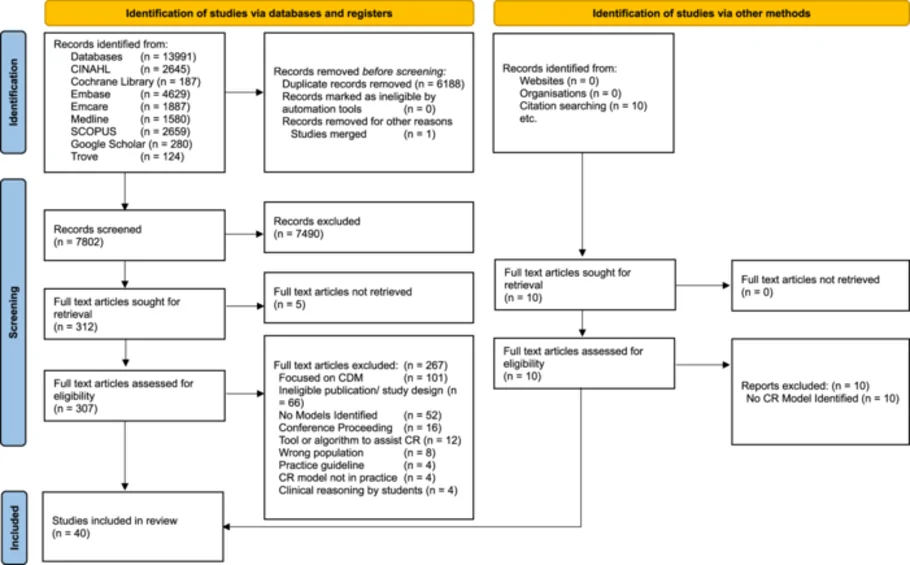
PRISMA flow chart.

### 3.2 Study Characteristics

Included studies were published between 1982 and 2023. Studies were predominantly conducted in Australia (*n* = 13), the United States (USA) (*n* = 9), and the United Kingdom (UK) (*n* = 5) (Table 2). Except for three surveys, all studies had a sample size between one and 33. Studies primarily focused on physiotherapists/manual therapists/physical therapists (*n* = 25) and occupational therapists (*n* = 11). One study each focused on podiatrists [37], psychologists [38], dietitians [39] and speech‐language pathologists [40]. No studies were found relating to social work or exercise physiology. Many studies utilized qualitative methodologies, though some also gathered quantitative data through surveys (*n* = 3) and mixed methods (*n* = 1).

**Table 2:** Characteristics of included studies.

Reference	Year	Country	Allied health professionals	Participant sample size	Study design	Data collection method	Setting
Ahlsen et al. [76]	2018	Norway	Physiotherapists	10 PTs	Narrative inquiry	Interviews	Private practice (MSK pain)
Ajjawi [77]	2006	Australia	Physiotherapists	12 PTs	Hermeneutic phenomenology	Observations, semi‐structured interviews,	Teaching Hospital, Private practice (1)
Ajjawi and Higgs [78]	2012	Australia	Physiotherapists	12 PTs	Hermeneutic phenomenology	Semi‐structured interviews, Observation, Written Exercises	Unspecified (cardiopulmonary, musculoskeletal or neurological Pt)
Alnervik and Svidén [51]	1996	Sweden	Occupational Therapists	5 OTs	Qualitative analysis	Videotaped observations, Interviews	University clinic (Medical rehab, neurologic rehab, rheumatology, and hand surgery)
Case et al. [43]	2000	UK	Physiotherapists	21 PTs	Qualitative analysis	Questionnaire	Hospital (cardiorespiratory)
Cheung et al. [60]	2018	Singapore	Occupational Therapists	14 OTs	Constructivist‐grounded theory	Individual in‐depth interviews	Hospital (UL RSI)
Copley et al. [46]	2008	Australia	Occupational Therapists	7 OTs	Qualitative analysis	In‐depth interviews, short questionnaires, and therapy observations	University clinic (pediatric learning difficulties)
Copley et al. [61]	2008	Australia	Occupational Therapists	1 OT	Qualitative Case Study	Observations, Documentation Review, Semi‐structured interviews	University clinic (UL hypertonicity management)
Cruz et al. [79]	2012	Portugal	Physiotherapists	4 PTs	Hermeneutic Phenomenology	Video films, semi‐structured interviews, patient charts, field notes	Unspecified (4 different practice settings with MSK expertise)
Curran et al. [37]	2006	UK	Podiatrists	8 Podiatrists	Qualitative analysis	Think aloud protocols	Hospital & NHS Podiatry clinics
Davies and Howell [80]	2012	USA	Physiotherapists	13 PTs	Qualitative analysis	Semi‐structured interviews, observations	Hospital, Private practice outpatient (acute LBP)
Doody and McAteer [64]	2002	Ireland	Physiotherapists	10 PTs	Qualitative analysis	Observations, audiotapes, semi‐structured interview	Hospital outpatient (n = 4), MSK Private Practice (n = 6),
Edwards et al. [2]	2004	Australia	Physiotherapists	6 PTs	Grounded Theory ‐ Multiple Case Studies	Observations, semi and unstructured interviews,	Private Practice, Domiciliary care, Community Health centers
Fleming [41]	1991	USA	Occupational Therapists	14 OTs	Phenomenology	Observations, Interaction, analysis of videotaped and transcribed treatment sessions, individual and group interviews	Teaching Hospital
Holdar et al. [81]	2013	Sweden	Physiotherapists	11 PTs	Phenomenography	Semi‐structured interviews, observations	Hospital Acute
Karvonen et al. [82]	2017	Finland	Physiotherapists	6 PTs	Qualitative analysis	Interviews	Outpatient Clinics (MSK/LBP)
Kristensen et al. [49]	2012	Denmark	Occupational Therapists	25 OTs	Hermeneutical phenomenology and Action Research	Field observations, Semi‐structured and short informal interviews, field notes, focus groups	Acute Hospital, Rehab Hospital, Public health centers (Neuro conditions)
Masley et al. [58]	2011	USA	Physiotherapists	18 PTs	Grounded Theory	Semi‐structured interviews	Acute Care
May et al. [47]	2008	UK	Physiotherapists	20 PTs	Delphi Study	Delphi survey three rounds	Primary care, Secondary care
May et al. [44]	2010	UK	Physiotherapists	9 PTs	Framework analysis	Semi‐structured interviews	NHS Trusts
McAllister et al. [40]	2020	Australia	Speech Therapists	4 SLTs	Exploratory	Videotaped observations, semi‐structured think‐aloud Interviews	Hospital Acute (metropolitan)
Milidonis et al. [63]	1999	USA	Physiotherapists	420 PTs	Practice analysis	Survey	Various Orthopedic Settings
Miller [65]	2009	Australia	Physiotherapists	19 PTs	Mixed Methods	Semi‐structured interviews	MSK Clinics
Nieto and Villarejo [38]	2020	Spain	Psychologists	45 Psychologists	Survey (Rational ‐ Experiential Inventory)	Online Survey	Spanish Public healthcare system
Noll et al. [55]	2001	USA	Physiotherapists	1 PT	Case Study	Observations, retrospective interviews	Orthopedic rehab
Paulazzo [50]	1992	USA	Occupational Therapists	118 OTs	Descriptive Study	Survey	Not Specified
Payton [42]	1985	USA	Physiotherapists	10 PTs	Descriptive Study	Structured interview, observations	Hospital, clinical, private practice
Plummer et al. [83, 84]	2005	Australia	Physiotherapists	14 PTs	Case Study	Think aloud protocol	Acute, Rehab, Research (Neurology)
Plummer et al. [48]	2006	Australia	Physiotherapists	33 PTs	Qualitative analysis	Focus groups and interviews	Healthcare and university departments
Rivett and Higgs [85]	1997	Australia	Physiotherapists	19 PTs	Qualitative analysis	Videotaped observations, Interviews	Manual therapy clinic
Rogers and Masagatani [57]	1982	USA	Occupational Therapists	10 OTs	Qualitative analysis	Observations, pre ax interviews, interview	Acute and diagnostic settings
Roine et al. [59]	2023	Finland	Physiotherapists	10 PTs	Qualitative analysis	Interviews	Finnish Primary Healthcare Centers
Rotor and Capio [86]	2018	Philippines	Physiotherapists	10 PTs	Descriptive phenomenology	Interviews	Filipino Hospital, Outpatient clinic, Community
Shafaroodi et al. [56]	2017	Iran	Occupational Therapists	15 OTs	Grounded Theory	Semi‐structured interviews	Mental and Physical Dysfunction Clinics
Sweeney and Doody [87]	2010	Ireland	Physiotherapists	12 PTs	Multiples Case Studies	Semi‐structured interviews	MSK Clinical Therapy
Thackray [54]	2014	UK	Physiotherapists	8 PTs	Qualitative analysis	Videotaped observations, Think aloud protocols, Interviews	Hospital
Unsworth [52]	2001	Australia	Occupational Therapists	5 OTs	Qualitative analysis	Headmounted videotapes	Rehabilitation
Unsworth [53]	2005	Australia	Occupational Therapists	13 OTs	Ethnography	Observations, video assisted recall	Rehabilitation
Vo et al. [39]	2021	Australia	Dietitians	11 Dietitians	Interpretive	Semi‐structured interviews, focus group	Hospital
Wainwright [45]	2006	USA	Physiotherapists	6 PTs	Grounded Theory	Observation, field notes, semi‐structured interviews	In‐patient rehabilitation

Abbreviations: MSK, musculoskeletal; OT, occupational therapists; PT, physiotherapists; SLT, speech therapist.

### 3.3 Description of Clinical Reasoning Models

Eleven models (Table 3) were identified across six Allied Health professions: hypothetico‐deductive reasoning (*n* = 25), pattern recognition (*n* = 19), three‐track reasoning (*n* = 5), dual‐process model (*n* = 3), dialectical model of reasoning (*n* = 1), systematic reasoning (*n* = 1), causal‐context‐intervening model (*n* = 1), listen‐try‐persevere model (*n* = 1), Masley et al.'s model for acute care (*n* = 1), reasoning model for initial assessment (*n* = 1), and understanding & negotiating model (*n* = 1). Included studies typically identified models by observations, interviews, and surveys, and deducing models used based on the data.

**Table 3:** Clinical reasoning models identified.

Reference	Allied health professionals	Hypothetico‐deductive (*n* = 25)	Pattern recognition (*n* = 19)	Three‐track reasoning (*n* = 5)	Dual‐process model (*n* = 3)	Single‐reported approaches (*n* = 1)
Ahlsen et al. [76]	Physiotherapists	X	X			
Ajjawi [77]	Physiotherapists	X	X			
Ajjawi and Higgs [78]	Physiotherapists	X				
Alnervik and Svidén [51]	Occupational Therapists			X		
Case et al. [43]	Physiotherapists		X			
Cheung et al. [60]	Occupational Therapists					Listen‐Try‐Persevere Model
Copley et al. [46]	Occupational Therapists		X			
Copley et al. [61]	Occupational Therapists					Understanding & Negotiating Model
Cruz et al. [79]	Physiotherapists	X				
Curran et al. [37]	Podiatrists	X	X			
Davies and Howell [80]	Physiotherapists	X	X			
Doody and McAteer [64]	Physiotherapists	X	X			
Edwards et al. [2]	Physiotherapists	X	X			Dialectical Model of Reasoning
Fleming [41]	Occupational Therapists	X	X			
Holdar et al. [81]	Physiotherapists	X	X			
Karvonen et al. [82]	Physiotherapists	X				
Kristensen et al. [49]	Occupational Therapists			X		
Masley et al. [58]	Physiotherapists					Masley et al.‘s model for acute care
May et al. [47]	Physiotherapists	X	X			
May et al. [44]	Physiotherapists	X				
McAllister et al. [40]	Speech Therapists	X	X			
Milidonis et al. [63]	Physiotherapists	X				
Miller [65]	Physiotherapists	X	X			
Nieto and Villarejo [38]	Psychologists				X	
Noll et al. [55]	Physiotherapists				X	
Paulazzo [50]	Occupational Therapists			X		
Payton [42]	Physiotherapists	X				
Plummer et al. [83, 84]	Physiotherapists	X	X			
Plummer et al. [48],	Physiotherapists	X	X			
Rivett and Higgs [85]	Physiotherapists	X				
Rogers and Masagatani [57]	Occupational Therapists					Reasoning model for initial assessment
Roine et al. [59]	Physiotherapists	X	X			Systematic
Rotor and Capio [86]	Physiotherapists	X				
Shafaroodi et al. [56]	Occupational Therapists					Causal‐Context‐Intervening Model
Sweeney and Doody [87]	Physiotherapists	X				
Thackray [54]	Physiotherapists	X	X		X	
Unsworth [52]	Occupational Therapists			X		
Unsworth [53]	Occupational Therapists			X		
Vo et al. [39]	Dietitians		X			
Wainwright [45]	Physiotherapists	X	X			

### 3.4 Hypothetico‐Deductive Reasoning

Appearing in 25 studies, the model most frequently reported was hypothetico‐deductive reasoning. Hypothetico‐deductive reasoning was most commonly used by physiotherapists (88%) though it was also reported among podiatrists [37], occupational therapists [41], and speech therapists [40]. Originally described in a medical setting, hypothetico‐deductive reasoning suggests that clinicians generate hypotheses based on patient information which are continually evaluated until sufficient data is required to satisfy a diagnosis [2, 42]. This model was widely recognized as being more thorough, less efficient, and better suited to novice clinicians than other diagnostic reasoning models [43, 44, 45]. Whilst hypothetico‐deductive reasoning was typically utilized during diagnosis, some studies found that it was also utilized in treatment planning and patient intervention [44].

### 3.5 Pattern Recognition

The second most common model of clinical reasoning was pattern recognition (*n* = 19) and was commonly observed being used alongside hypothetico‐deductive reasoning (16/19 occasions). Pattern recognition was found to be used by physiotherapists (*n* = 14), occupational therapists [41, 46], dietitians [39], speech therapists [40], and podiatrists [37]. Pattern recognition is categorized by rapid non‐analytical matching of a patient's presentation to patterns previously encountered by the clinician to form a diagnosis [2]. Studies showed pattern recognition was frequently employed by experienced clinicians who are able to draw from a larger base of clinical patterns [47]. Plummer et al. [48] suggested that pattern recognition is highly influenced by the initial observation, varies greatly between patients, and is less sequential than hypothetico‐deductive reasoning. Like the hypothetico‐deductive model, pattern recognition falls under diagnostic reasoning and reflects a biomedical model wherein clinical problems are objective and predictable [2].

### 3.6 Three‐Track Reasoning

First described by Fleming [26], three‐track reasoning was described in five studies with occupational therapists. Three‐track reasoning involves the rapid, ongoing, and dynamic shifting between procedural reasoning, interactive reasoning, and conditional reasoning [26]. Procedural reasoning focuses on the technical and biomedical aspects of care, interactive reasoning considers the clinician‐client relationship and meeting their unique needs, and conditional reasoning considers the patient's broader context and combines the former two [49, 50]. Alnervik and Svidén [51] sought to explore the interplay between the tracks and found that procedural was dominant while conditional reasoning was the least used. Three‐track reasoning is underlined by a less diagnostic approach than hypothetico‐deductive and pattern recognition and is instead more closely aligned with narrative reasoning which seeks to understand the client's unique contexts [52, 53].

### 3.7 Dual‐Process Theory

Dual‐process theory was cited in three studies and seeks to link two distinct systems of thinking: System one and System two. System one is characterized by fast, automatic, and often unconscious processes that rely on heuristics and pattern recognition, making it efficient for routine and familiar tasks, but prone to bias [38]. In contrast, System two involves slow, deliberate, and conscious processes that require logical reasoning and critical analysis. Although less susceptible to biases, System two is more resource‐intensive [38]. Whilst dual‐process theory as a concept originates from psychology, Thackray [54] and Noll et al. [55] both found evidence for its use in physiotherapy, particularly by linking system one to pattern recognition and system two to hypothetico‐deductive, they suggest clinical problems can be approached with one linking model. In both cases, as with Nieto and Villarejo's [38] survey of psychologists, dual‐process theory was identified as being a highly intuitive and typically implicit process.

### 3.8 Single‐Reported Approaches

Seven miscellaneous clinical reasoning models were identified. Edwards et al. [2] proposed a dialectical model among physiotherapists, wherein their reasoning shifts between the diagnostic process required to identify and manage client symptoms, and narrative processes required to understand and engage with client's experiences. Similarly, Shafaroodi et al. [56] tended towards the biopsychosocial model for occupational therapists with the causal‐context‐intervening model which describes the interconnected relationship between causal condition, client contexts, and intervening constructs (e.g., therapist beliefs and knowledge). The reasoning model for initial assessment [57] and the Masley et al. [58] model for acute care seek to describe models for specific points of care in occupational therapy and physiotherapy, respectively. Roine et al. [59] proposed a systematic model for physiotherapists, following a predefined framework to comprehensively examine all options without presumption and reduce the likelihood of missing important information. Cheung et al. [60] coined the listen‐try‐persevere model for occupational therapists educating clients with upper‐limb repetitive strain injury, by emphasizing the client‐centered nature of biopsychosocial care. Similarly, Copley, Turpin, et al. [61] found greater evidence of this relationship as a reasoning model with the understanding and negotiating model, which involves seeking client information, forming a hypothesis, then compensating with the client to by tailoring interventions to meet agreed goals.

## 4 Discussion

The purpose of this review was to explore and map the current body of literature on clinical reasoning models used by Allied Health professionals. This is the first review to comprehensively address the available evidence on clinical reasoning models across Allied Health professions. The findings indicate hypothetico‐deductive reasoning and pattern recognition were the most commonly reported models, while other models were reported less frequently across the remaining Allied Health professions included in this review.

The frequent reporting of hypothetico‐deductive reasoning and pattern recognition as clinical reasoning models highlights the necessity of accurate assessment and problem identification in effective clinical decision‐making. In one of the earliest descriptions of clinical reasoning models of physiotherapists, Payton [42] outlined a correlation between diagnostic accuracy and the richness of cue acquisition that precedes hypothesis generation in hypothetico‐deductive reasoning. His study aimed to replicate an investigation of clinical reasoning of physicians by Elstein et al. [62]. However, a survey of 420 physiotherapists [63] found that hypothetico‐deductive reasoning had been adapted to suit physiotherapy discipline. The widespread reporting of hypothetico‐deductive reasoning, particularly within physiotherapy, may be partly attributable to its broadly applicability across diverse clinical presentations. While diagnosis was described as its primary function, therapists also apply hypothetico‐deductive reasoning when selecting and implementing interventions [47, 64]. This broader application may help explain its use amongst Allied Health professions that are not primarily responsible for diagnosis, such as occupational therapy. A shift towards pattern recognition in the clinical reasoning of Allied Health professionals was linked to greater clinician experience [39, 43, 45, 65], as well as its fast and intuitive nature [54, 59]. However, pattern recognition was also demonstrated by novice clinicians [41, 64]. One mixed methods study suggested pattern recognition yielded greater diagnostic accuracy than hypothetico‐deductive, although the available data were limited [65].

While hypothetico‐deductive reasoning and pattern recognition are often associated with biomedical diagnostic practice, and narrative reasoning with person‐centered, biopsychosocial approaches, this association should not be read as a strict dichotomy. Diagnostic reasoning may be compatible with, and often necessary for, high‐quality biopsychosocial care; equally, the use of narrative reasoning does not by itself ensure that care is comprehensive or biopsychosocially informed. At the frontline practice, it is likely clinicians move fluidly between diagnostic, procedural, ethical, interactive, and narrative reasoning within a single encounter, often combining several modes to address a single clinical problem. What distinguishes a biopsychosocial approach is therefore less the type of reasoning used and more how these reasoning modes are integrated. That is whether diagnostic conclusions are situated within, rather than substituted for, an understanding of the patient's broader psychosocial context.

This integrated view is reflected across healthcare to move away from a biomedical to a biopsychosocial paradigm [66, 67], and is represented in some of the less‐cited clinical reasoning models. The causal‐context‐intervening model described by Shafaroodi et al. [56], the listen‐try‐persevere model [60] and the understanding and negotiating model [61] each account for individual patient factors and context, acknowledging health determinants beyond physical impairment [16]. That these models are identified only within occupational therapy is unsurprising, given narrative reasoning's predominance in the field, and its orientation towards the person's broader life context and meaning [68, 69]. Nonetheless, the commonly reported diagnostic reasoning models elsewhere in the literature does not inherently indicate an absence of narrative reasoning; rather, narrative reasoning tends to be described more broadly, outside the structured format of a defined clinical reasoning model. This may reflect the relative recency of the shift towards biopsychosocial practice (and its correspondingly limited description in the literature), or it may suggest that narrative reasoning, by nature more individualized and client‐centered, is less conducive to codification into systematic clinical reasoning models in the first place.

Our findings indicate that clinical reasoning research within Allied Health professions remains largely profession‐specific, with limited evidence exploring clinical reasoning approaches across different disciplinary contexts. No included study examined clinical reasoning models across multiple Allied Health professions, suggesting that the development and evaluation of clinical reasoning approaches have predominantly occurred within individual professional domains. Similar variability has been reported in the education of clinical reasoning across health disciplines. A scoping review of clinical reasoning curricula [32] found that there is a lack of uniformity in the teaching of clinical reasoning in tertiary education settings. The lack of collaborative research is particularly surprising given the increasing emphasis on multidisciplinary teamwork in healthcare [70, 71] and its demonstrated benefits for healthcare delivery and patient outcomes [21, 72]. This variability highlights the need for further investigation into how clinical reasoning approaches are developed, adapted, and applied across Allied Health practice and education. Given the complexity of contemporary healthcare, clinical reasoning approaches that incorporate diverse perspectives and consider the multifaceted nature of patient care may better reflect the realities of clinical practice. For example, integrating the narrative reasoning approach commonly described in occupational therapy with the diagnostic focus of physiotherapy‐based hypothetico‐deductive reasoning may offer opportunities to support more comprehensive approaches to clinical reasoning.

A notable finding of this review was that hypothetico‐deductive reasoning and pattern recognition were the most commonly reported clinical reasoning approaches, especially in physiotherapy, despite the emergence of alternative approaches, such as three‐track reasoning and dual‐process theory, within Allied Health education. Elvén et al. [32] conducted a scoping review of 26 studies examining clinical reasoning curricula in health professional education. They found that dual‐processing and script theory were the most frequently cited in curricula development and in explaining clinical reasoning processes, with the hypothetico‐deductive model mentioned less often [32]. This potentially highlights a disparity between the clinical reasoning models taught in educational settings and those actually employed in clinical practice, pointing to a broader education‐practice gap.

Such gaps are not uncommon in Allied Health, as illustrated by the case of biopsychosocial practice. Recent studies of physiotherapists have shown that although biopsychosocial practice is widely taught in physiotherapy curricula, its application and understanding in clinical practice is poor [73, 74]. These gaps likely stem from a range of factors, including ease of use, limited consultation time and prioritization of delivery of care. For instance, clinical reasoning models grounded in biopsychosocial practice such as the causal‐context‐intervening model, require careful consideration of each client's unique context. This may make them less practical than more structured, systematic models, such as pattern recognition, which can be applied efficiently and replicated quickly across different clients and settings.

### 4.1 Strengths and Limitations

This review was underpinned by established scoping review methodology and adhered to best practice standards for the conduct and reporting of scoping reviews. The research team comprised professionals from Allied Health disciplines, including physiotherapy, occupational therapy, and podiatry, ensuring that diverse perspectives were considered in line with the review's multidisciplinary focus. The inclusion of academic and gray literature increased the breadth of the included sources and limited publication and location bias. No date or country limits were placed to capture the full extent of clinical reasoning literature historically and internationally. However, as clinical reasoning definitions and terminology vary between Allied Health professions, despite a comprehensive search strategy, some publications may have been missed. The evidence base for this review was heavily skewed towards physiotherapy and occupational therapy, with comparatively limited literature available for podiatry, speech pathology, and dietetics, and no eligible studies identified for social work or exercise physiology. As such, the findings of this review may be most applicable to physiotherapy and occupational therapy, while its generalizability to other Allied Health professions, including those not represented in this review (such as radiography, optometry) remains to be established. The findings should be interpreted in the context of the methodological characteristics of the included studies. Many studies involved small samples, qualitative designs, or case‐based approaches, and methodological quality was not appraised. Therefore, the frequency of reporting of specific clinical reasoning approaches should not be interpreted as evidence of effectiveness, superiority, validity, or widespread uptake in clinical practice. Rather, these findings reflect patterns within the available literature and identify areas requiring further investigation. Finally, this review's eligibility criteria centered on structured clinical reasoning models, which largely reflect Western epistemological traditions, meaning Indigenous and other non‐Western frameworks for understanding and addressing health presentations (e.g. [75]) are likely underrepresented in this review.

### 4.2 Implications

This review has implications for Allied Health professions represented in this review, particularly practice and curricula development in physiotherapy and occupational therapy. The findings of this review highlight the potential need to further examine clinical reasoning education within these professions, particularly to ensure alignment between curricula and the evolving needs of clinical practice. Hypothetico‐deductive reasoning and pattern recognition being the most commonly reported in the literature is likely a reflection of what is intuitive and readily implementable for these allied health professionals. Given the increasing emphasis on a biopsychosocial approach to healthcare, the continued reporting of hypothetico‐deductive reasoning and pattern recognition warrants further investigation, including in Allied Health professions that are underrepresented in the current evidence base. If clinical reasoning models that align with a biopsychosocial approach are to be prioritized in the education and training of Allied Health professionals, consensus is needed on how this can be achieved in practice. This will require collaboration across sectors, including professional bodies, educational institutions and accreditation bodies, to ensure Allied Health practice remains contemporary and continues to meet the evolving needs of the community. Greater consideration of diverse clinical reasoning approaches may support more holistic allied health practice and better reflect the complexity of contemporary healthcare. Future research exploring clinical reasoning approaches across a broader range of Allied Health professions may help identify opportunities for developing more comprehensive models of clinical reasoning.

## 5 Conclusion

In total, 11 clinical reasoning models were identified in Allied Health practice from the literature, predominantly within physiotherapy and occupational therapy. Hypothetico‐deductive reasoning and pattern recognition were most commonly reported, while three‐track reasoning and dual‐processing theory were also frequently cited. Most of the literature on clinical reasoning models focused on physiotherapists and occupational therapists, with comparatively limited evidence available for psychology, podiatry, speech pathology, and dietetics, and no eligible studies identified for social work or exercise physiology. The findings indicate that, within the professions represented in this review, diagnostic reasoning models such as hypothetico‐deductive reasoning and pattern recognition continue to be more the most frequently reported than narrative models, despite pedagogical efforts to incorporate a broader range of clinical reasoning approaches. Furthermore, there was limited evidence of clinical reasoning models being developed across different Allied Health professions, which may not fully reflect the diverse and complex contexts in which Allied Health professionals typically practise. These findings highlight opportunities to further explore the teaching and application of clinical reasoning approaches within the Allied Health professions represented in this review, as well as the need for research examining approaches used by Allied Health professions not represented in the current evidence base. Further research involving Allied Health professionals, particularly those professions underrepresented in the current evidence base, can help to identify preferences and drivers influencing the use of diverse reasoning models in clinical practice, thereby supporting the integration of these models into contemporary Allied Health care.

## Author Contributions

**Henry Schultz:** conceptualization, writing – original draft, methodology, formal analysis, writing – review and editing. **Rosa Virgara:** writing – review and editing, conceptualization. **Ellen O'Callaghan:** supervision, writing – review and editing, conceptualization. **Ali Aguilar:** writing – review and editing, conceptualization. **Kristin Graham:** supervision, writing – review and editing, conceptualization. **Saravana Kumar:** supervision, methodology, conceptualization, writing – review and editing.

## Ethics Statement

Given the nature of the research reported in this manuscript (scoping review which summarizes existing literature), ethics approval was not required for this research.

## Conflicts of Interest

The authors declare no conflicts of interest.

## Transparency Statement

Henry Schultz affirms that this manuscript is an honest, accurate, and transparent account of the study being reported; that no important aspects of the study have been omitted; and that any discrepancies from the study as planned (and, if relevant, registered) have been explained. All authors have read and approved the final version of the manuscript, and all authors had full access to all the data in this study and take complete responsibility for the integrity of the data and the accuracy of the data analysis.

## Data Availability

The authors confirm that the data supporting the findings of this study are available within the manuscript and its supplementary materials.

## 1

**Appendix A.** Search syntaxes for published literature.

Search syntax for MEDLINE

**Table:**

#	Query	Results (25/1/24)
1	Allied Health Personnel/	13,202
2	Allied Health Occupations/	612
3	Occupational Therapists/	762
4	Occupational Therapy/	15,126
5	Physical Therapists/	3310
6	Podiatry/	2419
7	Speech Therapy/	6946
8	Speech‐Language Pathology/	3772
9	Social Work/	16,435
10	Social Workers/	1151
11	Dietetics/	8291
12	Nutritionists/	1812
13	Psychology/	24,192
14	Exercise Therapy/	50,262
15	(“allied health” or “allied health professional*“ or “allied health worker*“ or “allied health practitioner*“ or “allied health personnel*“ or “allied health occupation*“ or “occupational therapist*“ or physiotherapist* or “physical therapist*“ or podiatrist* or “speech therapist*“ or “speech pathologist*“ or “speech language pathologist*“ or “social worker*“ or nutritionist* or dietician* or dietitian* or psychologist* or “exercise physiologist*“ or “exercise therapist*“).ti,ab, kf.	84,293
16	1 or 2 or 3 or 4 or 5 or 6 or 7 or 10 or 11 or 12 or 13 or 14 or 15	191,118
17	Clinical Reasoning/	555
18	Clinical Decision Making/	14,766
19	(“clinical* reason*“ or “clinical judgment*“ or “clinical problem solving” or “clinical rationale” or “clinical decision making” or “professional reasoning” or “diagnostic reasoning” or “therapeutic reasoning” or “theoretical reasoning” or “narrative reasoning” or “interactive reasoning” or “collaborative reasoning” or “procedural reasoning” or “teaching reasoning” or “ethical reasoning”). ti, ab, kf.	39,429
20	17 or 18 or 19	51,472
21	16 and 20	1580

*Search syntax for Ovid EMBASE*

**Table:**

#	Query	Results (25/1/24)
1	paramedical personnel/	17,351
2	occupational therapy/	30,147
3	occupational therapist/	9534
4	physiotherapy/	118,762
5	physiotherapist/	29,877
6	podiatry/	3121
7	podiatrist/	1031
8	speech therapy/	18,233
9	speech language pathologist/	3975
10	social work/	31,692
11	social worker/	17,762
12	dietetics/	7651
13	dietitian/	18,435
14	psychology/	249,917
15	psychologist/	19,759
16	exercise physiology/	1448
17	(“allied health” or “allied health professional*“ or “allied health worker*“ or “allied health practitioner*“ or “allied health personnel*“ or “allied health occupation*“ or “occupational therapist*“ or physiotherapist* or “physical therapist*“ or podiatrist* or “speech therapist*“ or “speech pathologist*“ or “speech language pathologist*“ or “social worker*“ or nutritionist* or dietician* or dietitian* or psychologist* or “exercise physiologist*“ or “exercise therapist*“).ti,ab, kf.	136,174
18	or/1‐17	576,828
19	clinical reasoning/	1547
20	clinical decision making/	70,961
21	(“clinical* reason*“ or “clinical judgment*“ or “clinical problem solving” or “clinical rationale” or “clinical decision making” or “professional reasoning” or “diagnostic reasoning” or “therapeutic reasoning” or “theoretical reasoning” or “narrative reasoning” or “interactive reasoning” or “collaborative reasoning” or “procedural reasoning” or “teaching reasoning” or “ethical reasoning”). ti, ab, kf.	56,324
22	19 or 20 or 21	105,469
23	18 and 22	4629

*Search syntax for Ovid EMCARE*

**Table:**

#	Query	Results (25/1/24)
1	paramedical personnel/	3744
2	occupational therapy/	13,434
3	occupational therapist/	7480
4	physiotherapy/	35,524
5	physiotherapist/	12,257
6	podiatry/	1050
7	podiatrist/	794
8	speech therapy/	6038
9	speech language pathologist/	3537
10	social work/	20,353
11	social worker/	10,998
12	dietetics/	2271
13	dietitian/	5882
14	psychology/	24,551
15	psychologist/	9696
16	exercise physiology/	804
17	(“allied health” or “allied health professional*“ or “allied health worker*“ or “allied health practitioner*“ or “allied health personnel*“ or “allied health occupation*“ or “occupational therapist*“ or physiotherapist* or “physical therapist*“ or podiatrist* or “speech therapist*“ or “speech pathologist*“ or “speech language pathologist*“ or “social worker*“ or nutritionist* or dietician* or dietitian* or psychologist* or “exercise physiologist*“ or “exercise therapist*“).ti,ab, kf.	69,327
18	or/1‐17	159,229
19	clinical reasoning/	877
20	clinical decision making/	17,521
21	(“clinical* reason*“ or “clinical judgment*“ or “clinical problem solving” or “clinical rationale” or “clinical decision making” or “professional reasoning” or “diagnostic reasoning” or “therapeutic reasoning” or “theoretical reasoning” or “narrative reasoning” or “interactive reasoning” or “collaborative reasoning” or “procedural reasoning” or “teaching reasoning” or “ethical reasoning”). ti, ab, kf.	20,703
22	19 or 20 or 21	31,428
23	18 and 22	1887

*Search syntax for SCOPUS*

**Table:**

#	Query	Results (25/1/24)
1	(TITLE‐ABS‐KEY (“allied health” OR “allied health professional*“ OR “allied health worker*“ OR “allied health practitioner*“ OR “allied health personnel*“ OR “allied health occupation*“ OR “occupational therapist*“ OR physiotherapist* OR “physical therapist*“ OR podiatrist* OR “speech therapist*“ OR “speech pathologist*“ OR “speech language pathologist*“ OR “social worker*“ OR nutritionist* OR dietician* OR dietitian* OR psychologist* OR “exercise physiologist*“ OR “exercise therapist*“))	207,887
2	(TITLE‐ABS‐KEY (“clinical* reason*“ OR “clinical judgment*“ OR “clinical problem solving” OR “clinical rationale” OR “clinical decision making” OR “professional reasoning” OR “diagnostic reasoning” OR “therapeutic reasoning” OR “theoretical reasoning” OR “narrative reasoning” OR “interactive reasoning” OR “collaborative reasoning” OR “procedural reasoning” OR “teaching reasoning” OR “ethical reasoning”))	113,052
3	1 AND 2	2659

*Search syntax for CINAHL*

**Table:**

#	Query	Results (25/1/24)
S1	(MH (“Allied Health Personnel” OR “Allied Health Professions” OR “Occupational Therapy” OR “Occupational Therapists” OR “Physical Therapy” OR “Physical Therapists” OR Podiatry OR Podiatrists OR “Speech Therapy” OR “Speech‐Language Pathologists” OR “Social work” OR “Social workers” OR Dietetics OR Dietitians OR Nutritionists OR Psychology OR Psychologists OR “Exercise Physiology” OR “Exercise Physiologists”)	61,095
S2	(TI (“allied health” OR “allied health professional*“ OR “allied health worker*“ OR “allied health practitioner*“ OR “allied health personnel*“ OR “allied health occupation*“ OR “occupational therapist*“ OR physiotherapist* OR “physical therapist*“ OR podiatrist* OR “speech therapist*“ OR “speech pathologist*“ OR “speech language pathologist*“ OR “social worker*“ OR nutritionist* OR dietician* OR dietitian* OR psychologist* OR “exercise physiologist*“ OR “exercise therapist*“) OR AB (“allied health” OR “allied health professional*“ OR “allied health worker*“ OR “allied health practitioner*“ OR “allied health personnel*“ OR “allied health occupation*“ OR “occupational therapist*“ OR physiotherapist* OR “physical therapist*“ OR podiatrist* OR “speech therapist*“ OR “speech pathologist*“ OR “speech language pathologist*“ OR “social worker*“ OR nutritionist* OR dietician* OR dietitian* OR psychologist* OR “exercise physiologist*“ OR “exercise therapist*“)	76,908
S3	S1 OR S2	113,634
S4	(MH (“Clinical reasoning” OR “Decision making, Clinical” OR “Diagnostic Reasoning”))	39,030
S5	(TI (“clinical* reason*“ OR “clinical judgment*“ OR “clinical problem solving” OR “clinical rationale” OR “clinical decision making” OR “professional reasoning” OR “diagnostic reasoning” OR “therapeutic reasoning” OR “theoretical reasoning” OR “narrative reasoning” OR “interactive reasoning” OR “collaborative reasoning” OR “procedural reasoning” OR “teaching reasoning” OR “ethical reasoning”) OR AB (“clinical* reason*“ OR “clinical judgment*“ OR “clinical problem solving” OR “clinical rationale” OR “clinical decision making” OR “professional reasoning” OR “diagnostic reasoning” OR “therapeutic reasoning” OR “theoretical reasoning” OR “narrative reasoning” OR “interactive reasoning” OR “collaborative reasoning” OR “procedural reasoning” OR “teaching reasoning” OR “ethical reasoning”))	16,644
S6	S4 OR S5	50,921
S7	S3 AND S6	2,645

*Search syntax for Cochrane Library*

**Table:**

#	Query	Results (25/1/24)
#1	MeSH descriptor: [Allied Health Personnel] this term only	352
#2	MeSH descriptor: [Allied Health Occupations] this term only	8
#3	MeSH descriptor: [Occupational Therapy] this term only	1033
#4	MeSH descriptor: [Physical Therapists] this term only	218
#5	MeSH descriptor: [Podiatry] this term only	53
#6	MeSH descriptor: [Speech Therapy] this term only	424
#7	MeSH descriptor: [Social Workers] this term only	41
#8	MeSH descriptor: [Dietetics] this term only	126
#9	MeSH descriptor: [Nutritionists] this term only	71
#10	MeSH descriptor: [Psychology] this term only	400
#11	MeSH descriptor: [Exercise Therapy] this term only	13,880
#12	((allied NEXT health OR allied NEXT health NEXT professional* OR allied NEXT health NEXT worker* OR allied NEXT health NEXT practitioner* OR allied NEXT health NEXT personnel* OR allied NEXT health NEXT occupation* OR occupational NEXT therapist* OR physiotherapist* OR physical NEXT therapist* OR podiatrist* OR speech NEXT therapist* OR speech NEXT pathologist* OR speech NEXT language NEXT pathologist* OR social NEXT worker* OR nutritionist* OR dietician* OR dietitian* OR psychologist* OR exercise NEXT physiologist* OR exercise NEXT therapist*)):ti, ab, kw	21,201
#13	(OR #1‐#12)	35,743
#14	MeSH descriptor: [Clinical Reasoning] this term only	22
#15	MeSH descriptor: [Clinical Decision‐Making] this term only	516
#16	((clinical* NEXT reason* OR clinical NEXT judgment* OR clinical NEXT problem NEXT solving OR clinical NEXT rationale OR clinical NEXT decision NEXT making OR professional NEXT reasoning OR diagnostic NEXT reasoning OR therapeutic NEXT reasoning OR theoretical NEXT reasoning OR narrative NEXT reasoning OR interactive NEXT reasoning OR collaborative NEXT reasoning OR procedural NEXT reasoning OR teaching NEXT reasoning OR ethical NEXT reasoning)):ti, ab, kw	4154
#17	(OR #14‐#16)	4154
#18	#13 AND #17	187

**Appendix B.** Search syntaxes for gray literature.

*Search syntax for Google Scholar*

(“clinical reasoning models” OR “models of clinical reasoning”) AND allied health

Search limited to exclude patents and citations

Total: 280 results

*Search syntax for TROVE*

(“clinical reasoning” OR “clinical decision making”) AND “allied health”

Search limited to theses.

Total: 124

**Appendix C.** Data Extraction Form Template with examples.

**Table:**

Authors	Year	Title	Country	Setting	Sample Size	Study Design	Data Collection Method	AHP	Name of CRM(s)	Features of CRM(s)
Ahlsen et al. [76]	2018	Physiotherapists as detectives: Investigating clues and plots in the clinical encounter	Norway	Private practice (MSK pain)	10 PTs	Narrative inquiry	Interviews	Physiotherapists	Hypothetico‐deductive, Pattern Recognition	**Hypothetico Deductive “**By presenting the physiotherapists’ interpretation work as consisting of clue production, plot building and continuous weighing of different scenarios, our research resonates with Doode and McAteer's work on clinical reasoning in physiotherapy, which finds clinical reasoning to be a continuing and cyclical process of cue acquisition, hypothesis generation and evaluation of both.” **Pattern Recognition** “Our findings regarding physiotherapists’ construction of plots resonate with research suggesting that the recognition of patterns acquired through clinical experiences plays a role in clinical reasoning.” Does not go into any further detail about models.
Shafaroodi et al. [56]	2017	Occupational therapists’ clinical reasoning: A qualitative study	Iran	Mental and Physical Dysfunction Clinics	15 OTs	Grounded Theory	Semi‐structured interviews	Occupational Therapists	Causal‐Context‐Intervening Model	Influenced by BPS Model. Describes the interconnected relationship between causal condition (client's beliefs, knowledge, social aspects, economic aspects etc), client context (where they present e.g., hospital or outpatient clinic), and intervening construct (therapist beliefs, knowledge) and impact on therapist perspective. In turn influences action/interaction strategies (e.g., communication, organization, undertaken intervention) which are purposeful or deliberate acts which shape a phenomenon in some way. These acts include communication, organization and planning, undertaking the intervention. Outcomes of which are monitored and fed back to the client. Finally, by undertaking these tasks, it results in the final consequence which is an observable change. Authors also highlight some facilitating (knowledge, skills and experience of therapists) and constraining factors (dominance of medical perspective and lack of health insurance ‐ which means some treatment options were not funded) for clinical reasoning.

**Preferred Reporting Items for Systematic reviews and Meta‐Analyses extension for Scoping Reviews (PRISMA‐ScR) Checklist.**

**Table:**

SECTION	ITEM	PRISMA‐ScR CHECKLIST ITEM	REPORTED ON PAGE #
**TITLE**			
Title	1	Identify the report as a scoping review.	1
**ABSTRACT**			
Structured summary	2	Provide a structured summary that includes (as applicable): background, objectives, eligibility criteria, sources of evidence, charting methods, results, and conclusions that relate to the review questions and objectives.	2
**INTRODUCTION**			
Rationale	3	Describe the rationale for the review in the context of what is already known. Explain why the review questions/objectives lend themselves to a scoping review approach.	3
Objectives	4	Provide an explicit statement of the questions and objectives being addressed with reference to their key elements (e.g., population or participants, concepts, and context) or other relevant key elements used to conceptualize the review questions and/or objectives.	3
**METHODS**			
Protocol and registration	5	Indicate whether a review protocol exists; state if and where it can be accessed (e.g., a Web address); and if available, provide registration information, including the registration number.	5
Eligibility criteria	6	Specify characteristics of the sources of evidence used as eligibility criteria (e.g., years considered, language, and publication status), and provide a rationale.	5
Information sources*	7	Describe all information sources in the search (e.g., databases with dates of coverage and contact with authors to identify additional sources), as well as the date the most recent search was executed.	7
Search	8	Present the full electronic search strategy for at least 1 database, including any limits used, such that it could be repeated.	28
Selection of sources of evidence†	9	State the process for selecting sources of evidence (i.e., screening and eligibility) included in the scoping review.	7
Data charting process‡	10	Describe the methods of charting data from the included sources of evidence (e.g., calibrated forms or forms that have been tested by the team before their use, and whether data charting was done independently or in duplicate) and any processes for obtaining and confirming data from investigators.	7
Data items	11	List and define all variables for which data were sought and any assumptions and simplifications made.	7
Synthesis of results	13	Describe the methods of handling and summarizing the data that were charted.	8
**RESULTS**			
Selection of sources of evidence	14	Give numbers of sources of evidence screened, assessed for eligibility, and included in the review, with reasons for exclusions at each stage, ideally using a flow diagram.	8
Characteristics of sources of evidence	15	For each source of evidence, present characteristics for which data were charted and provide the citations.	11
Results of individual sources of evidence	17	For each included source of evidence, present the relevant data that were charted that relate to the review questions and objectives.	13
Synthesis of results	18	Summarize and/or present the charting results as they relate to the review questions and objectives.	14
**DISCUSSION**			
Summary of evidence	19	Summarize the main results (including an overview of concepts, themes, and types of evidence available), link to the review questions and objectives, and consider the relevance to key groups.	16
Limitations	20	Discuss the limitations of the scoping review process.	19
Conclusions	21	Provide a general interpretation of the results with respect to the review questions and objectives, as well as potential implications and/or next steps.	19
**FUNDING**			
Funding	22	Describe sources of funding for the included sources of evidence, as well as sources of funding for the scoping review. Describe the role of the funders of the scoping review.	20

JBI = Joanna Briggs Institute; PRISMA‐ScR = Preferred Reporting Items for Systematic reviews and Meta‐Analyses extension for Scoping Reviews.

* Where *sources of evidence* (see second footnote) are compiled from, such as bibliographic databases, social media platforms, and Web sites.

† A more inclusive/heterogeneous term used to account for the different types of evidence or data sources (e.g., quantitative and/or qualitative research, expert opinion, and policy documents) that may be eligible in a scoping review as opposed to only studies. This is not to be confused with *information sources* (see first footnote).

‡ The frameworks by Arksey and O'Malley (6) and Levac and colleagues (7) and the JBI guidance (4, 5) refer to the process of data extraction in a scoping review as data charting.

§ The process of systematically examining research evidence to assess its validity, results, and relevance before using it to inform a decision. This term is used for items 12 and 19 instead of “risk of bias” (which is more applicable to systematic reviews of interventions) to include and acknowledge the various sources of evidence that may be used in a scoping review (e.g., quantitative and/or qualitative research, expert opinion, and policy document).

*From:* Tricco AC, Lillie E, Zarin W, O'Brien KK, Colquhoun H, Levac D, et al. PRISMA Extension for Scoping Reviews (PRISMAScR): Checklist and Explanation. Ann Intern Med. 2018;169:467–473. doi: 10.7326/M18‐0850.
